# WIC and non-WIC Infants and Children Differ in Usage of Some WIC-Provided Foods

**DOI:** 10.1093/jn/nxy157

**Published:** 2018-08-31

**Authors:** Joanne F Guthrie, Diane J Catellier, Emma F Jacquier, Alison L Eldridge, Wendy L Johnson, Anne C Lutes, Andrea S Anater, Erin E Quann

**Affiliations:** 1 Economic Research Service, US Department of Agriculture, Washington, DC; 2 RTI International, Research Triangle Park, NC; 3 Nestlé Research, Vers-chez-les-Blanc, Lausanne, Switzerland; 4 Nestlé Nutrition/Gerber Products Company, Florham Park, NJ

**Keywords:** nutrition, WIC, breastfeeding, food consumption patterns, Feeding Infants and Toddlers Study

## Abstract

**Background:**

USDA's Special Supplemental Nutrition Program for Women, Infants, and Children (WIC) provides expert-chosen supplemental foods to improve the diets and health of low-income infants and children <5 y of age, but dietary behaviors of WIC participants are not well characterized.

**Objective:**

The purpose of this analysis was to examine differences in food consumption patterns between WIC participants and nonparticipants.

**Methods:**

FITS 2016 is a nationwide cross-sectional study of children <4 y (*n* = 3235). Data were weighted to provide US population–representative results. Children were categorized as WIC participants or nonparticipants, with the latter divided into lower- and higher-income nonparticipants. Group differences were assessed via the Wald test (demographics) and Rao-Scott modified chi-square test (breastfeeding prevalence). Differences in percentage consuming WIC-provided and selected other foods between WIC participants and nonparticipants were evaluated with the use of ORs and 95% CIs.

**Results:**

WIC infants were less likely to breastfeed than were higher-income nonparticipants at 0–5.9 mo (45% compared with 74%) and less likely than both nonparticipant groups at 6–11.9 mo (30% compared with 49–60%). WIC 6- to 11.9-mo-olds were more likely to consume infant cereals and vegetables than were lower-income nonparticipants. WIC 12 to 23.9-mo-olds were more likely to drink whole milk (which WIC provides at this age) than were nonparticipants (72% compared with 59–64%), whereas WIC participants 24–47.9 mo were more likely to drink low- and nonfat milks (which WIC provides at this age) than were nonparticipants (45% compared with 13–22%). WIC participants 6–47.9 mo were more likely to drink juice than were nonparticipants.

**Conclusions:**

Continued improvements in early dietary patterns are warranted for WIC and non-WIC children. Breastfeeding among WIC participants is a continuing challenge. Findings suggest that baby-food cereals, vegetables, and fruits (all provided by WIC) contribute importantly to WIC infants’ diets, whereas WIC children are more likely to use lower-fat milks after 2 y of age than are non-WIC participants.

## Introduction

**FIGURE 1:**
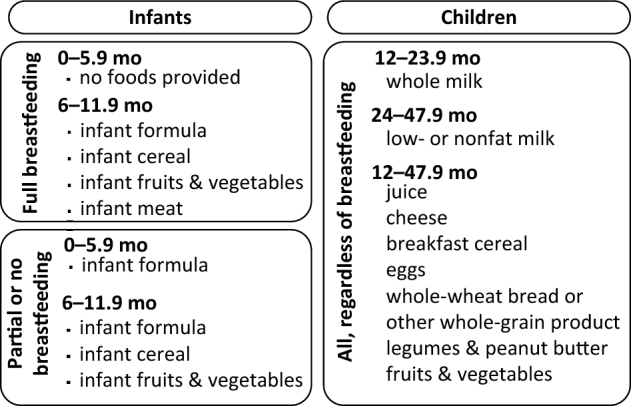
WIC food packages for infants and children (based on information in reference 1). WIC, Special Supplemental Nutrition Program for Women, Infants, and Children.

The Special Supplemental Nutrition Program for Women, Infants, and Children (WIC) provides low-income, nutritionally at-risk pregnant, breastfeeding, and postpartum women, infants, and children <5 y with packages of supplemental foods selected to meet their nutritional needs (**Figure 1**). WIC participants also receive nutrition education and referrals to health care and social services. Breastfeeding promotion remains a key area of focus given lower rates of breastfeeding among WIC participants compared to the national average. In fiscal year 2015, WIC served 1.9 million women, 1.9 million infants, and 4.2 million children each month at a total cost of $6.2 billion (2). WIC serves more than half of US infants under 1 y and more than a quarter of 1–5-y-olds (1).

 

In 2009, the WIC food packages were updated for the first time since the program started to better align with the latest nutrition knowledge. Juice was removed from the infant food packages, and baby-food fruit and vegetable options were added; low- or nonfat milk became the standard choice for children starting at 2 y. Amounts of juice, eggs, and milk were reduced, and a cash-value voucher to purchase fruits and vegetables was added to children's packages.

In 2014, the USDA commissioned an expert committee of the National Academies of Sciences, Engineering, and Medicine (NASEM) to review the impact of the 2009 WIC food packages and make recommendations for changes if needed. The committee's review (3) relied heavily on small studies that used state or local data owing to the paucity of national data.

This study aims to provide national consumption data from the 2016 Feeding Infants and Toddlers Study (FITS) and compare infant feeding and child food consumption behaviors of WIC participants with lower- and higher-income nonparticipants.

This is a cross-sectional, descriptive analysis that explores differences in dietary patterns between WIC and non-WIC infants and children. These results are intended to inform future trend analysis and suggest areas for further research. For this reason, and because of the many potential confounding factors (such as self-selection into WIC), the reader is cautioned against drawing definitive conclusions about the causal effects of WIC participation on infant feeding practices or children's food choices.

## Methods

### FITS survey methods

The FITS 2016 is a nationwide, cross-sectional study of parents/caregivers of children from birth to 4 y living in the 50 states and Washington DC. The FITS 2016 builds on the 2 previous FITS surveys conducted in 2002 and 2008 (4, 5). Data were collected from 4 sampling frames designed to cover the US population, and the resulting sample was weighted and calibrated to the US 2014 Census divisions, accounting for child age, WIC status, race/ethnicity, and educational attainment of the parent or caregiver, with the use of the Statistical Analysis System (version 9.1.3, SAS Institute, Cary, NC, 2014). Stratified random sampling with targeted oversampling was used to achieve prespecified sample size targets for age and WIC participants (*n* = 1161) and nonparticipants (*n* = 2068). Full details of the survey methodology are available elsewhere in this Supplement (6).

The survey included a recruitment questionnaire consisting of sociodemographic and lifestyle (e.g., screen time, physical activity) questions, a feeding practices questionnaire, and one or two 24-h dietary recalls. All study instruments were pilot tested before use and were available in English and Spanish. The final instruments were reviewed and approved by the institutional review boards of RTI International, the University of Minnesota Nutrition Coordinating Center, and the Docking Institute of Public Affairs, Fort Hays State University, who assisted with data collection in the recruitment phase. Data were collected from June 2015 to May 2016.

Demographic characteristics and WIC participation were derived from the recruitment questionnaire. Past WIC participation and length of WIC participation were not assessed, nor were differences in state- or clinic-level WIC policies and practices. WIC foods consumed and breastfeeding status were derived from the feeding practices questionnaire responses.

Specific foods eaten were derived from the 24-h dietary recalls (*n* = 3235), which were collected via telephone by trained interviewers from the University of Minnesota's Nutrition Coordinating Center using the Nutrition Data System for Research (NDSR, version 2015, University of Minnesota, Minneapolis, MN). A second 24-h recall was collected from a random subsample of 25% of the total sampled population (*n* = 799, of which 275 were participating in the WIC program).

All foods and beverages reported in the 24-h dietary recalls were assigned to food groups through the use of a food group classification scheme aligned with the USDA’s What We Eat in America (7) and, to the extent possible, the FITS 2008 food classification system (8); the FITS 2016 food database has also been updated to reflect consumption habits and products available in 2016.

### Statistical analysis

Participation in WIC for all analyses presented here was based on the child's reported participation in WIC at the time of the dietary interview. Participants missing this information (*n* = 6) were dropped from the analysis. Respondents who reported that the child did not participate in WIC were divided into 2 subgroups: lower-income (and likely WIC-eligible) nonparticipants (*n* = 641), and higher-income (and likely WIC-ineligible) nonparticipants (*n* = 1427) (9). Income data were collected categorically. To approximate eligibility, a continuous income variable was imputed from the categoric data with the use of a percentile-constrained inverse-cumulative density function method (10). Missing income data were imputed from education level. The continuous income variable, reported household size, and the 2016–2017 WIC eligibility cutoffs (11) were used to assign nonparticipants to 1 of the 2 income groups. Because this approach considers only imputed (not actual) income and does not consider nutritional risk, those groups are labeled as “lower-income” and “higher-income” nonparticipants, rather than WIC eligible and ineligible, to avoid confusion. Further methodological details for the WIC eligibility approximation are provided in the Supplemental Methods.

Demographic differences between the WIC participant group and the lower- and higher-income nonparticipant groups were estimated via the Wald test; differences in breastfeeding status were estimated via the Rao-Scott modified chi-square test (SUDAAN, release 9, Research Triangle Institute, Research Triangle Park, NC, 2005), which corrects the chi-square test for use with data from a complex sample survey (12, 13). Significance for these comparisons was evaluated with the use of an alpha of 0.05.

Food consumption patterns, specifically the percentage of children in each WIC status group that consumed food from each food group, were derived from the 24-h dietary recall data. These data were further stratified by age range into younger infants (0–5.9 mo), older infants (6–11.9 mo), and young children (12–47.9 mo). The descriptive findings reflect the unadjusted prevalence of infant feeding patterns and consumption of foods by WIC and non-WIC infants and children. Because this is an exploratory analysis (i.e., we did not start with a specific hypothesis) to assess whether consumption patterns differed between WIC and non-WIC groups, we do not present results of formal hypothesis tests (i.e., *P* values). Differences between higher- and lower-income nonparticipants were not assessed. We did, however, identify food categories for children >6 mo (the age at which WIC begins providing foods other than infant formula) with large differences in the percentage consuming between WIC and non-WIC groups and where the 95% CI for the OR does not contain the null value of 1. To reduce the risk of spurious relations, we limited the food categories examined for differences to foods provided by WIC (e.g., baby-food fruits, vegetables) and a small number of other foods of interest (e.g., we examine consumption of all fruits to gain more insight into the role of baby-food fruits).

## Results

### Sample characteristics

**Supplemental Table 1** summarizes sample sizes and characteristics for the study subgroups. Almost 40% of WIC participant caregivers had no more than a high school education. More than half (54%) were also receiving Supplemental Nutrition Assistance Program (SNAP) benefits.

### Foods obtained from WIC

WIC provides different food packages based on the child's age and breastfeeding status (Figure 1). If mothers do not exclusively breastfeed infants 0–5.9 mo, WIC provides infant formula but no other foods. Packages for infants 6–11.9 mo and children 1–5 y include foods chosen to meet nutritional shortfalls. Three-quarters of WIC participants 0–5.9 mo old received formula (**Table 1**). Among WIC participants 6–11.9 mo old, the most commonly reported WIC food eaten was infant cereals (76%), followed by baby-food fruits, infant formula, and baby-food vegetables (68–70%). Only 16% of WIC 6- to 11.9-mo-olds were reported to eat baby-food meats from WIC; however, WIC provides baby-food meats only to breastfed infants, which account for only 30% of WIC participants in this age group. Among children 12–47.9 mo, milk was the most popular item, followed by fruits. Whole-wheat grains, eggs, cheese, breakfast cereals, and vegetables were at roughly similar levels of popularity, eaten by 60–66% of children.

**TABLE 1:** WIC foods reported consumed by infants and children participating in WIC^1^

Infants 0–11.9 mo		Children 12–47.9 mo	
Food	Consuming, %	Food	Consuming, %
Infants 0–5.9 mo (*n* = 600)		Children 12–47.9 mo (*n* = 1728)	
Infant formula^2^	75	Milk^3^	87
Infants 6–11.9 mo (*n* = 901)		Fruits	75
Infant cereal	76	Whole-wheat grains^4^	66
Baby-food fruit	70	Eggs	66
Infant formula	69	Cheese	66
Baby-food vegetables	68	Breakfast cereal	61
Fruits (non–baby-food)^5^	27	Vegetables	60
Vegetables (non–baby-food)^5^	17	Peanut butter	48
Baby-food meat^6^	16	Legumes	42

1 Values are percentages consuming WIC food, based on feeding practices questionnaire item “What foods does [child] eat from WIC?” For each age range, only foods that might be provided by WIC to that age are shown. WIC, Special Supplemental Nutrition Program for Women, Infants, and Children.

2 Although infant formula is the only food available from WIC for infants <6 mo, use of other foods was reported for some younger infants (not shown). It may be that responders misunderstood the question and reported use of these foods from any purchase source.

3 Includes cow milk, soy milk, other.

4 Includes whole-wheat bread and other whole-grain foods.

5 Whether WIC provides vouchers for non–baby-food fruits and vegetables to 6- to 11.9-mo-olds is up to the individual states.

6 Only provided in breastfeeding package.

### Prevalence of breastfeeding

**FIGURE 2:**
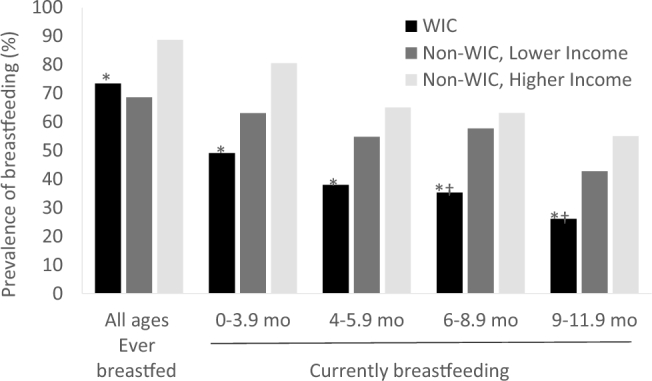
Percentage of mothers who ever breastfed or currently breastfeed infants ≤12 mo by WIC participation, from FITS 2016 feeding practices questionnaire. *WIC significantly different than higher-income (likely not WIC-eligible) nonparticipants, *P *< 0.05; ^†^WIC significantly different than lower-income (likely WIC-eligible) nonparticipants, *P *< 0.05; based on Rao-Scott modified chi-square test. WIC, Special Supplemental Nutrition Program for Women, Infants, and Children.

WIC participants were significantly less likely to have ever breastfed (i.e., initiated breastfeeding, regardless of whether they were still breastfeeding at the time of the survey) than higher-income nonparticipants (**Figure 2**), but compared with lower-income nonparticipants, the difference was nonsignificant. From 0–5.9 mo, WIC participants were significantly less likely to be currently breastfeeding than higher-income nonparticipants, and although fewer reported breastfeeding than lower-income nonparticipants, that difference was smaller and nonsignificant. From 6–11.9 mo, WIC participants were significantly less likely to breastfeed than were higher- and lower-income nonparticipants.

**FIGURE 3:**
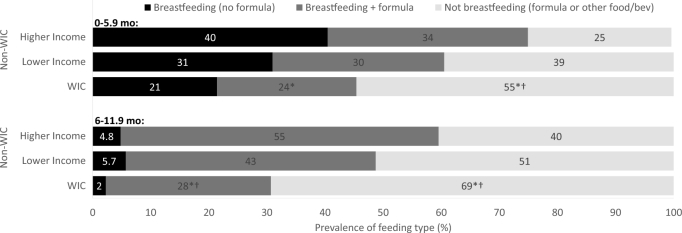
Percentage of mothers exclusively or partially breastfeeding infants 0–5.9 and 6–11.9 mo by WIC participation status. *WIC significantly different than higher-income (likely not WIC-eligible) nonparticipants, *P *< 0.05; ^†^WIC significantly different than lower-income (likely WIC-eligible) nonparticipants, *P *< 0.05; based on Rao-Scott modified chi-square test. WIC, Special Supplemental Nutrition Program for Women, Infants, and Children.

Some mothers who breastfed supplemented with formula (partial breastfeeding) and some did not (exclusive breastfeeding). In both cases, they may also have fed complementary foods, such as infant cereal; if they were not feeding any formula, that was considered exclusive breastfeeding, regardless of what other foods and beverages were consumed. Regardless of WIC participation status, mothers of infants 0–5.9 mo who breastfed were about equally likely to breastfeed exclusively as to supplement breastfeeding with formula (**Figure 3**). Thus, the overall lower prevalence of breastfeeding by WIC participants reflects lower prevalence in both exclusive breastfeeding and partial breastfeeding. Compared with higher-income nonparticipants, WIC participants were less likely to exclusively breastfeed and less likely to partially breastfeed. Compared with lower-income nonparticipants, the differences were nonsignificant. Mothers of infants 6–11.9 mo who breastfed mostly supplemented with formula and very few breastfed exclusively, regardless of WIC participation group (Figure 3). The prevalence of exclusive breastfeeding was too low (<6%) to see any significant differences between WIC participants and nonparticipants, but WIC participants were significantly less likely to partially breastfeed than both were higher- and lower-income nonparticipants.

### Complementary food consumption for younger infants (0–5.9 mo)

Complementary foods are defined as solid foods and liquids other than breast milk or formula that are introduced during the first year of life. WIC does not provide such foods to infants <6 mo old. Few infants 0–3.9 mo consumed infant cereal (the only food consumed by an appreciable number of infants in this age group), but at 4–5.9 mo, there was a notable increase in the percentage of infants reporting complementary food consumption, mostly infant cereal, fruit and 100% juice, and vegetables (**Table 2**). No notable differences were seen between WIC participants and nonparticipants.

**TABLE 2:** Consumption of complementary foods, infants aged 0–3.9 and 4–5.9 mo, by WIC participation^1^

	Children consuming, %							
	Age 0–3.9 mo				Age 4–5.9 mo			
			Nonparticipants				Nonparticipants	
Food category	All (*n* = 305)	WIC participants (*n* = 121)	Lower income^2^ (*n* = 54)	Higher income^2^ (*n* = 130)	All (*n* = 295)	WIC participants (*n* = 124)	Lower income^2^ (*n* = 50)	Higher income^2^ (*n* = 121)
Milk (not breast milk or formula)^3^	1.8	1.8	1.5	3.0	2.0	0.7	5.7	3.2
Milk products^4^	1.2	1.2	0	2.0	2.7	2.4	7.4	1.2
Grains	14	15	21	8.9	54	60	46	43
Infant cereal^5^	13	15	19	6.9	50	56	43	40
Fruits and fruit juices	8.3	7.1	13	8.6	39	42	52	29
Solid fruit^6^	6.5	6.4	7.6	6.1	37	39	51	28
100% juice^7^	4.2	3.5	8.3	4.0	5.5	4.8	10	4.8
Vegetables^8^	2.9	1.5	5.2	4.8	34	35	39	31
Meats/proteins^9^	1.7	1.2	1.4	3.0	4.3	3.6	7.1	4.5

1 Values are mean percentages of children consuming the food category during a single 24-h recall. WIC, Special Supplemental Nutrition Program for Women, Infants, and Children.

2 Lower-income nonparticipants are likely WIC eligible; higher-income nonparticipants are likely not WIC eligible. See the Methods section for further details.

3 Includes cow milk, plant-based substitutes, and goat milk, but excludes breast milk and infant formula.

4 Includes cheese and yogurt.

5 Includes any kind of infant cereal, regardless of grain (i.e., rice, oat, quinoa, wheat, multigrain, or unknown grain).

6 Includes both baby-food fruit and non–baby-food fruit; excludes 100% juice.

7 Includes both baby 100% juice and non–baby 100% juice.

8 Includes baby-food vegetables, non–baby-food vegetables, and white potatoes.

9 Includes meats, poultry, fish, legumes, nuts, and seeds, but does not include cheese or yogurt.

### Food consumption for older infants (6–11.9 mo)

**Table 3** provides results for children ≥6 mo. Because a few differences for 6- to 11.9-mo-olds are masked by the 6-mo interval used in Table 3, **Supplemental Table 2** provides results for older infants in 3-mo intervals (6–8.9 mo, 9–11.9 mo). ORs and 95% CIs were not calculated for these smaller age groupings.

**TABLE 3:** Consumption of WIC and selected other foods, by WIC status, for children aged 6–11.9, 12–23.9, and 24–47.9 mo^1^

			Non-WIC participants			
Food group	All	WIC participants	Lower income^2^	OR (95% CI)^3^	Higher income^2^	OR (95% CI)^3^
6–11.9 mo^4^						
Any grain product	84	85	80	—	84	—
Infant cereal^5^ (WIC food)	52	56	38	2.11 (1.37, 3.24)	50	1.29 (0.91, 1.81)
Any fruit^6^	74	73	79	—	74	—
Baby-food fruit^7^ (WIC food)	49	56	48	1.39 (0.92, 2.12)	36	2.30 (1.63, 3.25)
Non–baby-food fruit^8^	36	28	49	0.40 (0.26, 0.62)	49	0.40 (0.28, 0.58)
100% juice^9^	27	34	22	1.79 (1.10, 2.92)	15	2.88 (1.86, 4.45)
Any vegetable^10^	72	74	61	1.76 (1.12, 2.77)	72	1.06 (0.73, 1.57)
Baby-food vegetables^11^ (WIC food)	45	55	32	2.61 (1.69, 4.02)	30	2.84 (2.00, 4.04)
Non–baby-food vegetables^12^	38	31	38	0.76 (0.49, 1.16)	52	0.43 (0.30, 0.61)
Meats/proteins^13^	38	38	41	—	37	—
Baby-food meats (WIC food, exclusively breastfed only)	4.2	5.5	3.5	—	1.8	—
Sweets, desserts^14^	30	31	31	—	26	—
Sugar-sweetened beverages^15^	8.6	10	11	0.88 (0.41, 1.88)	4	2.67 (1.22, 5.87)
12–23.9 mo^16^						
Any cow milk	83	84	83	—	81	—
Whole milk (WIC food)	67	72	59	1.73 (1.14, 2.65)	64	1.43 (1.00, 2.03)
Cheese (WIC food)	36	32	41	0.71 (0.46, 1.08)	39	0.76 (0.53, 1.08)
Grains, grain products	95	96	93	—	96	—
Whole-grain products^17^ (WIC food)	69	70	62	1.42 (0.93, 2.19)	71	0.93 (0.65, 1.34)
Family cereal^18^	54	58	49	1.42 (0.94, 2.14)	50	1.37 (0.97, 1.91)
Family cereal, not presweetened (WIC food)	30	33	24	1.56 (0.99, 2.47)	31	1.09 (0.76, 1.56)
Any fruit^6^ (WIC food)	77	69	80	0.55 (0.34, 0.88)	87	0.33 (0.21, 0.50)
100% juice^9^ (WIC food)	50	63	51	1.60 (1.06, 2.41)	32	3.56 (2.50, 5.05)
Any vegetable^10^ (WIC food)	72	71	72	0.94 (0.59, 1.49)	75	0.82 (0.56, 1.21)
Meats/proteins^13^	83	84	82	—	82	—
Eggs (WIC food)	27	29	27	1.10 (0.70, 1.72)	25	1.19 (0.82, 1.72)
Legumes (WIC food)	9.9	8.6	8.5	1.01 (0.49, 2.06)	12	0.68 (0.40, 1.17)
Peanut butter (WIC food)	14	13	13	0.97 (0.85, 1.15)	14	0.90 (0.54, 1.50)
Sweets, desserts^14^	69	70	70	—	68	—
Sugar-sweetened beverages^15^	29	34	34	1.00 (0.65, 1.53)	20	2.03 (1.38, 2.99)
24–47.9 mo^19^						
Any cow milk	81	81	85	—	78	—
Reduced fat (2%)	34	18	37	—	43	—
Low- or nonfat milk (WIC food)	27	45	13	5.36 (2.40, 12.0)	22	2.92 (1.76, 4.85)
Cheese (WIC food)	40	36	34	1.07 (0.58, 1.98)	46	0.67 (0.39, 1.12)
Grains, grain products	95	95	94	—	95	—
Whole-grain products^17^ (WIC food)	59	51	56	0.81 (0.45, 1.46)	67	0.51 (0.31, 0.85)
Family cereal^18^	52	52	60	0.70 (0.39, 1.27)	49	1.12 (0.67, 1.85)
Family cereal, not presweetened (WIC food)	26	30	22	1.51 (0.73, 3.15)	24	1.32 (0.76, 2.29)
Any fruit^6^ (WIC food)	78	70	76	0.73 (0.38, 1.40)	84	0.43 (0.23, 0.79)
100% juice^9^ (WIC food)	47	63	42	2.26 (1.24, 4.14)	38	2.74 (1.62, 4.64)
Any vegetable^10^ (WIC food)	73	69	78	0.64 (0.32, 1.24)	74	0.81 (0.45, 1.45)
Meats/proteins^13^	88	91	83	—	88	—
Eggs (WIC food)	25	27	17	1.82 (0.93, 3.55)	27	1.01 (0.56, 1.80)
Legumes (WIC food)	9.5	7.8	9.6	0.79 (0.23, 2.67)	11	0.71 (0.24, 2.13)
Peanut butter (WIC food)	17	13	14	0.91 (0.39, 2.09)	20	0.56 (0.26, 1.21)
Sweets, desserts^14^	83	81	87	—	81	—
Sugar-sweetened beverages^15^	45	43	65	0.40 (0.22, 0.73)	38	1.22 (0.74, 2.03)

1 Values are mean percentages of children consuming the food category during a single 24-h recall, unless otherwise indicated. WIC, Special Supplemental Nutrition Program for Women, Infants, and Children.

2 Lower-income nonparticipants are likely WIC eligible; higher-income nonparticipants are likely not WIC eligible. See the Methods section for further details.

3 Values are ORs for the percentage of WIC participants consuming compared with the percentage consuming for the higher- or lower-income nonparticipant group, and the 95% CIs around the ORs. —, the differences between groups were small enough that we did not estimate OR or CI.

4 Total *n* for All, WIC participants, Lower income, and Higher income groups = 901, 375, 169, and 357, respectively.

5 Includes any kind of baby-food cereal, regardless of grain (i.e., rice, oat, quinoa, wheat, multigrain, or unknown grain).

6 Includes any kind of fruit, whether baby food or not; excludes 100% juice.

7 Includes commercial and homemade pureed baby-food fruit and non–baby-food fruit; excludes 100% juice.

8 Includes any fruit that is not baby food; excludes 100% juice.

9 Includes any 100% fruit juice regardless of whether it is specifically labeled for babies or not. Beverages that are <100% fruit juice are included in sugar-sweetened beverages.

10 Includes dark green, orange, red, starchy, and other vegetables, whether baby food or not, as well as white potatoes.

11 Includes commercial and homemade pureed baby-food vegetables.

12 Includes non–baby-food dark green, orange, red, starchy, and other vegetables, as well as white potatoes; excludes baby food.

13 Includes meats, poultry, fish, legumes, nuts, and seeds, but does not include cheese or yogurt.

14 Includes sweet baked goods, cereal and nutrition bars, candy, ice cream and other frozen desserts, jellies and jams, milk flavorings, and baby-food desserts and cookies.

15 Includes soft drinks, fruit-flavored drinks, tea and coffee, and sports drinks. Excludes 100% fruit juice.

16 Total *n* for All, WIC participants, Lower income, and Higher income groups = 1132, 380, 233, and 519, respectively.

17 Includes 100% whole-grain foods and whole-grain–rich foods that are 50–99% whole grains.

18 Includes any ready-to-eat or hot cereal that is not infant cereal.

19 Total *n* for All, WIC participants, Lower income, and Higher income groups = 596, 161, 135, and 300, respectively.

#### Milk and milk products

Fewer than 5% of infants 6–8.9 mo consumed non–baby milks (i.e., milks other than infant formula or breast milk), and among 9- to 11.9-mo-olds, 17% overall and

 

 

15% of WIC infants consumed non–baby milks (Supplemental Table 2).

#### Grains

About three-quarters (77%) of all 6–8.9-mo-olds and most 9–11.9-mo-olds (91%) were eating ≥1 item from the grain group (Supplemental Table 2). WIC infants 6–11.9 mo were more likely to consume infant cereals than were lower-income non-WIC infants (Table 3).

#### Fruits and 100% juice

About three-quarters of infants 6–11.9 mo consumed solid fruit, with no difference between WIC and other groups (Table 3). However, WIC participants were more likely to eat baby-food fruits and less likely to eat non–baby-food fruits than the higher-income nonparticipant group. WIC participants were more likely to drink 100% juice than either nonparticipant group.

#### Vegetables

Similarly, about three-quarters of 6- to 11.9-mo-olds consumed a vegetable on the day of the recall, and WIC participants were more likely to eat a vegetable than were lower-income nonparticipants (Table 3). WIC participants 6–11.9 mo were also more likely to eat baby-food vegetables than either comparison group and less likely to eat non–baby-food vegetables than the higher-income nonparticipant group.

#### Meats and other proteins

More than one-third (38%) of 6- to 11.9-mo-olds consumed some type of meat or protein food (Table 3), but few (<5%) consumed baby-food meats. There were no differences between WIC participants and either nonparticipant group.

#### Sweets and sugar-sweetened beverages

Among 6- to11.9-mo-olds, 30% consumed a sweet food, with little difference among groups (Table 3). Less than 9% consumed a sugar-sweetened beverage (SSB; note that SSBs do not include 100% fruit juice). WIC participants were more likely to consume SSBs than were higher-income nonparticipants.

### Food consumption for young children (12–47.9 mo)

#### Milk and milk products

On the day of the survey, 83% of 12–23.9-mo-olds and 81% of 24–47.9-mo-olds consumed some type of milk (Table 3). WIC provides whole milk for children aged 12–24 mo and low- or nonfat milk for children >24 mo. About two-thirds (67%) of 12–23.9-mo-olds consumed whole milk, with more WIC participants consuming it than lower-income non-WIC children. At 24–47.9 mo, reduced-fat (2%) milk is the most common milk choice overall, consumed by 34% of children; but compared with both non-WIC groups, more WIC children consumed low-fat (1%) and nonfat milk. Less than half of 12–23.9- and 24–47.9-mo-olds consumed cheese, with WIC children no different from the 2 comparison groups in likelihood of consumption.

#### Grains

Almost all young children 12–47.9 mo (95%) consumed some type of grain on the survey day (Table 3). At least 1 type of whole-grain product was consumed by 69% of children 12–23.9 mo and 59% of children 24–47.9 mo. At 24–47.9 mo, fewer WIC participants consumed some type of whole-grain product than higher-income nonparticipants. The likelihood of WIC children consuming breakfast cereals that were not presweetened did not differ from that of other groups.

#### Fruits and fruit juices

Among 12–23.9-mo-olds, fewer WIC children consumed fruit, compared with both lower- and higher-income nonparticipants (Table 3). Among 24–47.9-mo-olds, WIC children differed only from the higher-income non-WIC children, with 70% of WIC children consuming fruit, compared with 84% of higher-income non-WIC children. At both 12–23.9 mo and 24–47.9 mo, more WIC participants consumed 100% juice than both nonparticipant groups.

#### Vegetables

More than one-quarter of all children 12–23.9 and 24–47.9 mo did not consume a vegetable on the recall day, with no differences among groups.

#### Meats, other proteins

Of the foods in this category, WIC provides nonmeat proteins in the form of eggs and dried beans/legumes or peanut butter. For children 12–23.9 and 24–47.9 mo, consumption of eggs was around 25% and consumption of dried peas, beans, and legumes was around 10% across all WIC status groups. Consumption of peanut butter was around 15% with no differences across groups.

#### Sweets, SSBs, and savory snack foods

Sixty-nine percent of all 12–23.9-mo-olds and 83% of all 24–47.9-mo-olds consumed a sweet or dessert (excluding SSBs) on the day of the recall, with WIC consumption, at 70% and 81% respectively, similar to that of the overall group (Table 3). SSBs were consumed by 29% of children 12–23.9 mo and by 50% of children 24–47.9 mo. Among younger children, more WIC participants consumed SSBs than higher-income nonparticipants, whereas among older children, fewer WIC participants consumed SSBs compared with lower-income non-WIC children.

## Discussion

The large nationwide sample and detailed dietary collection provided by FITS 2016 offer some of the most comprehensive information available on dietary patterns of WIC-participating infants and toddlers after the WIC food package changes implemented in 2009. Findings provide information on how well participants’ dietary behaviors compare with WIC program objectives and where improvements are still needed. As a descriptive exploratory analysis, it also suggests promising directions for future research. However, limitations that hinder our ability to infer a causal relation between intakes described here and the WIC program must be acknowledged (see the Limitations section).

### Key findings: infants (<12 mo)

Findings on prevalence of breastfeeding are consistent with USDA administrative data (14) and support the NASEM expert committee's conclusion that further efforts are needed to promote breastfeeding initiation and continuation among WIC participants (3). The NASEM report recommends promotion of partial breastfeeding as a strategy to increase breastfeeding rates, along with research to identify further strategies for increasing breastfeeding initiation and continuation among WIC participants. The FITS 2016 data indicate that partial breastfeeding is a widely used strategy, especially between 6 and 12 mo. However, it appears to be less common among WIC mothers. An enhanced WIC package for mothers who partially breastfeed may enhance promotion of partial breastfeeding, as suggested by NASEM, and make the choice to continue breastfeeding more feasible for WIC mothers, potentially encouraging breastfeeding through the first year of life.

The FITS 2016 data indicate the overwhelming majority of WIC and other infants are not consuming complementary foods before 4 mo; more than half of infants between 4 and 6 mo are consuming some form of solid food. USDA's WIC Infant and Toddlers’ Feeding Practices Study II had similar findings (15) and notes that the small number introducing solids before 4 mo is a considerable improvement compared with findings from its first WIC feeding practices study conducted in 1994–1995. Further investigation of reasons for introduction of complementary foods between 4 and 6 mo could be helpful to WIC nutrition educators.

Between 6 and 12 mo, the infant begins the transition into a diet that adds other foods to breast milk or formula, with introduced foods serving the dual purpose of meeting immediate needs and initiating healthful eating habits. Fruits and vegetables are under-consumed by most Americans, children and adults (7). Findings suggest WIC's provision of baby-food vegetables supports inclusion of these foods in participating children's diets. Although FITS 2008 (16) found that WIC infants were less likely to eat vegetables than were nonparticipants, in FITS 2016, 6- to 11.9-mo-old WIC infants were more likely to consume vegetables than were lower-income nonparticipants (Table 3). The WIC infants were more likely to consume baby-food vegetables than were other infants, suggesting the important role that baby-food vegetables play in their diets. There was no difference between WIC infants and both groups of non-WIC infants in likelihood of consuming fruit from all sources, but more WIC infants ate baby-food fruit than higher-income nonparticipants. WIC's provision of baby-food fruit through 12 mo may be important to WIC infants’ having the same likelihood of fruit consumption as other infants. At the same time, 27% of WIC 6- to 11.9-mo-olds did not consume fruit on the survey day, and 26% did not consume a vegetable, suggesting further improvement is needed.

The 2017 NASEM report recommends offering the option to substitute a fruit and vegetable cash-value voucher for part or all of the baby-food fruits and vegetables provided by WIC to determine if it helps facilitate greater consumption overall. It will be important to investigate the effectiveness of this approach and related issues, such as the need for accompanying nutrition education promoting nutritious but under-consumed options such as dark green and orange vegetables, as well as age-appropriate guidance (e.g., fruits and vegetables with the correct size and texture to avoid posing a choking hazard) (3).

Iron is a critical nutrient for infants and young children, and iron-fortified infant cereals, iron-fortified formula (for formula-fed infants), and iron-rich meats are important sources. FITS 2008 showed that infant cereal was the top food source of iron and 9 other vitamins and minerals for 6- to11.9-mo-olds (17). Among those infants who did not consume infant cereal, a substantial proportion (30–40%) had iron intakes below the Estimated Average Requirement compared with those infants who ate infant cereal (<8%). The higher prevalence of reported consumption of infant cereals among 6- to 11.9-mo-old WIC infants, compared with their low-income non-WIC counterparts, is therefore an encouraging finding. Similar to non-WIC infants, <6% of WIC-participating infants consume baby-food meats, which are only provided through the breastfeeding package (so, to ∼30% of WIC participants in this age group). The NASEM report (3) has noted the lack of acceptance of baby-food meats and has suggested considering reduction of baby-food meats. It may be worth exploring why mothers do not feed baby-food meats and considering strategies for encouraging their intake—for example, manufacturers may be able to develop products that are more appealing, whereas nutrition educators may need to further emphasize early needs for iron.

### Key findings: children 12–47.9 mo

Among 12–47.9-mo-olds, the most notable difference between the diets of WIC participants and other children is their shift from whole milk to low- or nonfat cow milk beginning at 24 mo. This is consistent with WIC package rules, and WIC respondents report that milk is the item in the child package they are most likely to use. Non-WIC children also shift away from whole milk at 24 mo but are more likely to shift to 2% reduced-fat milk.

As with infants, there was a major focus on encouraging children to eat more fruits and vegetables as part of the 2009 WIC food package changes, which added a cash-value voucher specifically for purchase of fruits and vegetables, while still providing fruit juice as part of child packages. WIC participants in 2016 are about equally likely to consume vegetables as are nonparticipants. Just as addition of baby-food vegetables to the infant package may have promoted infants’ vegetable intake, so too the addition of the cash-value voucher may have promoted children's vegetable intake. Nevertheless, as with other children, a substantial minority of WIC children did not eat a vegetable on the survey day. The NASEM expert committee's recommendation to increase the value of the cash-value voucher (3) may further promote vegetable intake by WIC children.

Among 12–23.9-mo-olds, WIC participants were less likely to eat fruit than either comparison group, whereas at 24–47.9 mo, they differed only from higher-income children. Across both age groups, WIC children were more likely to drink fruit juice than were their non-WIC counterparts. The NASEM committee (3) has recommended that child packages contain less juice and participants be given the option of choosing to receive an extra $3 as part of their fruit and vegetable cash-value voucher instead of juice. This may help to shift WIC children's juice and fruit consumption in line with expert recommendations to limit juice in favor of solid fruit (18).

Along with previous support for consumption of iron-rich unsweetened cereals, the 2009 WIC package sought to promote consumption of whole grains. National purchasing data indicate that WIC participants have increased whole grain purchasing since the package was changed to encourage it (19). Whole-grain breads and cereals have become more widely available and consumed in the past decade (20), which may have led to changes in the comparison groups as well. Increased information on the benefits of whole grains may have particularly affected behavior of the better-educated higher-income comparison group, who were more likely to have consumed any whole-grain food than WIC participants among children 24–47.9 mo. Nevertheless, >30% of children consumed no whole-grain item on the survey day. The NASEM committee recommendation that all breakfast cereals available to children through WIC be whole-grain rich may further improve whole grain consumption by WIC participants (3).

For both WIC participants and nonparticipants, energy-dense, low-nutrient sweets were more prevalent in the diets of older age groups. This trend to more routine consumption of foods and beverages that nutritionists recommend for “occasional” consumption continues through school age (21) and may contribute to obesity or displace healthier options. Associations between WIC participation and usage of these items were inconsistent across age groups, with WIC-participating infants 6–11.9 mo and children 12–23.9 mo more likely to consume SSBs than higher-income nonparticipants, whereas WIC children 24–47.9 mo were less likely to consume SSBs than were lower-income nonparticipants. Population-wide efforts to discourage early introduction of these foods and beverages in exchange for healthier options may complement WIC's efforts to improve the diets of young children.

### Strengths

FITS 2016 is a national study based on the use of the automated multiple-pass method by trained interviewers, which is designed to minimize self-reporting errors and is consistent with USDA data. WIC participants were oversampled in the study to allow for sufficient sample sizes to make comparisons between WIC participants and nonparticipants (6). Following the approach of Condon et al. (9), comparison groups were subdivided by income to aid interpretation.

These findings add to those obtained from other research conducted since the change in the WIC food package in 2009. The cross-sectional comparisons presented in this paper provide useful information on the food habits of WIC participants that can help WIC program managers and nutritionists to prioritize and develop nutrition education messages and to assess the potential benefits of program changes recommended by the NASEM report. WIC is the only USDA food assistance program for which nutrition education is a required element. Identification of dietary behaviors most in need of further improvement can guide development of nutrition education programs and materials. Thus, these findings further inform our understanding of WIC's role in improving the diets of infants and young children.

### Limitations

Because the FITS 2016 is a cross-sectional study, the findings cannot be used to draw conclusions regarding the causal effects of WIC. WIC participants differ from other groups on many observed factors besides income, as well as unobserved factors that may affect both food choices and their decision to participate in WIC (22, 23). Factors related to the decision to participate in WIC were not assessed. Selection bias, the bias that occurs when individuals choose to participate in—or “self-select” into—a program, is poorly understood in general and in the WIC program in particular (3). It is believed to occur for reasons that might bias results either positively (24) or negatively (25), and there is no consensus on methods to correct for WIC self-selection bias (3). In addition, about half of WIC participants also received SNAP benefits: thus, it is more difficult to attribute associations exclusively to WIC, because infant formula and other foods included in the WIC package can also be purchased via SNAP benefits.

Further, although WIC is a national program, some WIC policies vary by state, such as whether a cash-value voucher for fruits and vegetables could be substituted for baby foods in the package for infants 6–12 mo; however, we do not have information on such differences and therefore cannot explore their associations with intakes. The data set also does not identify past WIC participation of older children, which might have influenced their dietary behaviors.

Finally, self-reported dietary recalls are limited by the ability to remember and accurately report foods consumed. Because dietary information in this study was reported by parents or caregivers, it may not fully represent the actual intake of the child. However, use of the automated multiple-pass method by trained interviewers is designed to minimize this inaccuracy.

### Looking forward: considering how proposed changes in the WIC package could improve children's diets

The NASEM committee recommended other changes to the WIC food packages that would have the potential to improve children's diets (3). Notably, they recommended adding seafood to children's food packages by incorporating canned tuna and other oily fish into a 3-mo rotation with peanut butter and legumes. Few children in the FITS 2016 data set were eating these foods, suggesting that their inclusion in the package could benefit diets, but effective strategies to encourage acceptance may be a necessary complement.

To support reduction in children's intake of added sugars, the NASEM committee also encouraged eliminating the flavored milk option in the WIC package and reducing the limit on sugar content of yogurt. Very few state WIC agencies allow flavored milk (3), and the FITS 2016 data show that unflavored milk is a far more common choice, with WIC children not consuming flavored milk more frequently than other children their age (data not shown). About one-quarter of WIC children reported consuming yogurt. The NASEM committee cited food industry information indicating that lower-sugar yogurts should be available in the marketplace, making the requirement of lower-sugar yogurts feasible (3). Moreover, the change in program standards could incentivize development of more lower-sugar yogurt options and wider availability of such options in the retail marketplace. The precedent is shown by previous market reaction to WIC package rules: for example, manufacturers have reformulated breakfast cereals to meet WIC iron content standards (26), and more small food stores stocked whole-wheat bread after it was added to the WIC food packages for women and children in 2009 (27). Such changes can have positive spillover benefits for non-WIC consumers, increasing availability of healthy products. With increasing availability, these healthier products may appear a more normative choice, creating a virtuous cycle in which WIC participants more easily accept WIC foods and may be more likely to continue to consume them after they “graduate” from the program.

Future research will build on the findings of this paper. Plans include the use of the data from FITS 2008 (16) and 2016 to identify changes in food consumption patterns over time as the WIC food packages have evolved. However, differences in study methodology between 2008 and 2016 (including WIC subject groupings and the food classification scheme) make it necessary to harmonize the 2 data sets before robust comparisons can be made; this work is under way.

## Supplementary Material

Supplement Tables

## Notes

Published in a supplement to *The Journal of Nutrition*. Select contents of this supplement were presented at the Experimental Biology 2017 conference in a session titled, “Informing B-24 Dietary Guidelines: Findings from the New Feeding Infants and Toddlers Study 2016,” held in Chicago, IL, April 22, 2017. Supplement Coordinators were William Dietz, Milken School of Public Health at the George Washington University and Andrea S Anater, RTI International. The FITS research was funded by Nestlé Research (Lausanne, Switzerland) through a contract with RTI International and its subcontractor, the University of Minnesota. Dr. Dietz is a compensated consultant to RTI on this project. The article contents are the responsibility of the authors and do not necessarily represent the opinions or recommendations of Nestlé. Publication costs for this supplement were defrayed in part by the payment of page charges. This publication must therefore be hereby marked “advertisement” in accordance with 18 USC section 1734 solely to indicate this fact. The opinions expressed in this publication are those of the authors and are not attributable to the sponsors or the publisher, Editor, or Editorial Board of *The Journal of Nutrition*.

Supported by Nestlé Research, Lausanne, Switzerland.

Author disclosures: EFJ and ALE are employed by Nestlé Research (funder); EEQ and WLJ are employed by Nestlé Nutrition/Gerber Products Company. JFG, DJC, ACL, and ASA, no conflicts of interest.

The views expressed are those of the authors and are not those of the US Department of Agriculture or its Economic Research Service and should not be attributed to the US Department of Agriculture or its Economic Research Service.

Supplemental Tables 1 and 2 and Supplemental Methods are available from the “Supplementary data” link in the online posting of the article and from the same link in the online table of contents at https://academic.oup.com/jn/.

Abbreviations used:

FITS: Feeding Infants and Toddlers Study
NASEM: National Academies of Sciences, Engineering, and Medicine
SNAP: Supplemental Nutrition Assistance Program
SSB: sugar-sweetened beverage
WIC: Special Supplemental Nutrition Program for Women, Infants, and Children

## References

[1] Oliveira V, Frazão E The WIC program: background, trends, and economic issues, 2015 edition. Washington (DC): US Department of Agriculture, Economic Research Service; 2015.

[2] Oliveira V The food assistance landscape: FY 2015 annual report. US Department of Agriculture ERS, editor. 2016 Available from: https://www.ers.usda.gov/publications/pub-details/?pubid=44062

[3] National Academies of Sciences, Engineering, and Medicine (NASEM). Review of WIC food packages: improving balance and choice: final report. Washington (DC): The National Academies Press; 2017. 28605175

[4] Briefel RR, Kalb LM, Condon E, Deming DM, Clusen NA, Fox MK, Harnack L, Gemmill E, Stevens M, Reidy KC The Feeding Infants and Toddlers Study 2008: study design and methods. J Am Diet Assoc 2010;110(12 Suppl):S16–26. 21092765 10.1016/j.jada.2010.09.005

[5] Devaney B, Kalb L, Briefel R, Zavitsky-Novak T, Clusen N, Ziegler P Feeding Infants and Toddlers Study: overview of the study design. J Am Diet Assoc 2004;104:8–13. 10.1016/j.jada.2003.10.023 14702012

[6] Anater AS, Catellier DJ, Levine BA, Krotki KP, Jacquier EF, Eldridge AL, Bronstein KE, Harnack LJ, Lorenzana Peasley JM, Lutes AC The Feeding Infants and Toddlers Study (FITS) 2016: study design and methods. J Nutr 2018;148:1516S–24S. 29878140 10.1093/jn/nxy035 PMC6126632

[7] US Department of Agriculture (Agricultural Research Service). What we eat in America. Agricultural Research Service, editor. Beltsville, MD; 2016.

[8] Siega-Riz AM, Deming DM, Reidy KC, Fox MK, Condon E, Briefel RR Food consumption patterns of infants and toddlers: where are we now? J Am Diet Assoc 2010;110(12):S38–51. 21092767 10.1016/j.jada.2010.09.001

[9] Condon E, Drilea S, Lichtenstein C, Mabli J, Madden E, Niland K Diet quality of American young children by WIC participation status: data from the National Health and Nutrition Examination Survey, 2005–2008. Rockville, MD: Walter R. McDonald & Associates, Inc. and Mathematica Policy Research for the Food and Nutrition Service, US Department of Agriculture; 2015.

[10] Couzens GL, Peterson K, Berzofsk M Income interpolation from categories using a percentile-constrained inverse-CDF approach. Survey Practice 2016;9(4):0032.

[11] US Department of Agriculture (Food and Nutrition Service). WIC income eligibility guidelines 2016 [cited 2016]. Available from: https://www.fns.usda.gov/wic/wic-income-eligibility-guidelines.

[12] Rao J, Scott A The analysis of categorical data from complex sample surveys: chi-squared tests for goodness-of-fit and independence in two-way tables. J Am Stat Assoc 1981;76:221–30.

[13] Rao J, Scott A On chi-squared tests for multi-way tables with cell proportions estimated from survey data. Ann Stat 1984;12:46–60.

[14] Thorn B, Tadler C, Huret N, Trippe C, Ayo E, Mendelson M, Patlan K, Schwartz G, Tran V WIC participant and program characteristics 2014. US Department of Agriculture FaNS, editor. Alexandria, VA; 2015 Available from: https://www.fns.usda.gov/wic/wic-participant-and-program-characteristics-2014.

[15] May L, Borger C, Weinfield N, MacAllum C, DeMatteis J, McNutt S, Whaley S, Ritchie L, Sallack L WIC infant and toddler feeding practices study – 2: infant year report. Rockville, MD: Westat; 2017 Available from: https://www.fns.usda.gov/wic/wic-infant-and-toddler-feeding-practices-study-2-infant-year-report.

[16] Deming D, Briefel R, Reidy K Infant feeding practices and food consumption patterns of children participating in WIC. J Nutr Educ Behav 2014;46(3S):S29–37. 24809994 10.1016/j.jneb.2014.02.020

[17] Finn K, Callen C, Bhatia J, Reidy K, Bechard L, Carvalho R Importance of dietary sources of iron in infants and toddlers: lessons from the FITS study. Nutrients 2017;9:733. 10.3390/nu9070733 PMC5537847 28696361

[18] Heyman M, Abrams S Fruit juice in infants, children, and adolescents: current recommendations. Pediatrics 2017;139(6):e20170967. 28562300 10.1542/peds.2017-0967

[19] Oh M, Jensen H, Rahkovsky I Did revisions to the WIC program affect household expenditures on whole grains? Appl Econ Perspect Policy 2016;38(4):578–98.

[20] Mancino L, Kuchler F Demand for whole-grain bread before and after the release of the Dietary Guidelines. Appl Econ Perspect Policy 2012;34:76–101.

[21] Cole N, Fox M Diet quality of American school-age children by school lunch participation status: data from the National Health and Nutrition Examination Survey, 1999-2004. US Department of Agriculture FaNS, Office of Research, Nutrition and Analysis, editor. Alexandria, VA; 2008 Available from: https://www.fns.usda.gov/diet-quality-american-school-age-children-school-lunch-participation-status-data-national-health-and.

[22] National Research Council. Estimating eligibility and participation for the WIC program: final report. Washington (DC): The National Academies Press; 2003. 25057680

[23] Ponza M, Devaney B, Shackman G, Woelfel ML, Ziegler P, Reidy K, Squatrito C, Pruzek R, Stratton H Looking at WIC. J Am Diet Assoc 2004;104(7):1074. 10.1016/j.jada.2004.05.198 15215761

[24] Besharov D, Germanis P Rethinking WIC: an evaluation of the Women, Infants, and Children program. Washington (DC): AEI Press; 2001.

[25] Bitler M, Currie J Does WIC work? The effects of WIC on pregnancy and birth outcomes. J Policy Anal Manage 2005;24(1):73–91. 15584177 10.1002/pam.20070

[26] Oliveira V, Frazão E Painting a more complete picture of WIC: how WIC impacts nonparticipants. Amber Waves. 2015 Apr 6. Available from: https://www.ers.usda.gov/amber-waves/2015/april/painting-a-more-complete-picture-of-wic-how-wic-impacts-nonparticipants/.

[27] Andreyeva T, Luedicke J, Middleton A, Long M, Schwartz M Positive influence of the revised Special Supplemental Nutrition Program for Women, Infants, and Children food packages on access to healthy food. J Acad Nutr Diet 2012;112:850–8. 22709812 10.1016/j.jand.2012.02.019

