# Supplementary material for: WIC and non-WIC Infants and Children Differ in Usage of Some WIC-Provided Foods
Source: J Nutr. 2018 Aug 31;148(Suppl 3):1547S–1556S. doi: 10.1093/jn/nxy157 (PMC6126634; doi:10.1093/jn/nxy157)
Supplement: Supplement Tables [file nxy157_supplement_tables.docx]

# Online Supplemental Material

**Supplemental Methods**

**Details of Determining Probable WIC Eligibility of Non-participants**

Children under 5 y are eligible for WIC if their family’s income is below 185% of the federal poverty guidelines for their household size, and the child is at nutritional risk. A definitive determination of income eligibility for nonparticipants is not possible for two reasons:

1. Income data were collected categorically for FITS (e.g., $10,000-$19,999) rather than as a single specific value (e.g., $16,360), and the categories do not correspond exactly to the various WIC eligibility cut-points by household size.
2. Data on nutritional risk were not collected.

However, it is possible to approximate income eligibility by imputing a continuous income variable. First, if an income category was not reported, it was imputed from education level. Then, we used a percentile-constrained inverse-cumulative density function method ([28](#_ENREF_28)) to probabilistically assign a point value for income to each nonparticipant.

The imputed income, reported household size, and the 2016-2017 WIC eligibility cut-points ([29](#_ENREF_29)) were used to designate nonparticipants as likely income eligible or likely income ineligible. The approach enforces the correct relationship among income bracket, household size, and WIC income eligibility thresholds; for example, the lower-income non-participants reporting incomes over $50,000 (see Table 1) also reported household sizes between 5 (eligibility threshold is $52,614) and 8 (eligibility threshold is $75,647).

Because this approach approximates income and does not consider nutritional risk, those groups are labeled as “lower income” and “higher income” nonparticipants, rather than WIC eligible and ineligible, to avoid implying greater precision than we have.

Supplemental Table 1. FITS 2016: Selected sample characteristics for all respondents, WIC participants, and lower- and higher-income nonparticipants^1^

| Variable | All (n=3,229) ^3^ | WIC Participants (n=1,161) | Nonparticipants | | Overall  *P*-values^2^ |
| --- | --- | --- | --- | --- | --- |
|  |  |  | Lower income  (n=641) | Higher income (n=1,427) |  |
| **Child's Race/Ethnicity** |  | | | |  |
| Hispanic | 15±0.6 | 21±1.2 | 15±1.4 | 9.1±0.8 | <0.0001 |
| Non-Hispanic White | 67±0.8 | 54±1.5 | 62±1.9 | 80±1.1 | <0.0001 |
| Non-Hispanic Black | 14±0.6 | 22±1.2 | 19±1.5 | 5.4±0.6 | <0.0001 |
| Non-Hispanic Other | 4.6±0.4 | 3.7±0.6 | 3.9±0.8 | 5.7±0.6 | <0.0001 |
| **Respondent’s Education** |  | | | |  |
| < High school | 4.3±0.4 | 8.8±0.8 | 4.4±0.8 | 0.6±0.2 | <0.0001 |
| High school or equivalent | 19±0.7 | 31±1.4 | 26±1.7 | 6.8±0.7 | <0.0001 |
| Some college/postsecondary | 23±0.7 | 29±1.3 | 29±1.8 | 15±0.9 | <0.0001 |
| Completed college | 39±0.9 | 27±1.3 | 34±1.9 | 50±1.3 | <0.0001 |
| Some graduate work/degree | 15±0.6 | 4.6±0.6 | 6.2±1.0 | 28±1.2 | <0.0001 |
| **Household Income^4^** |  | | | |  |
| Less than $10,000 | 8.5±0.5 | 18±1.1 | 11±1.2 | 0.1±0.1 | <0.0001 |
| $10,000 to $19,999 | 9.3±0.5 | 18±1.1 | 15±1.4 | 0.2±0.1 | <0.0001 |
| $20,000 to $34,999 | 18±0.7 | 31±1.4 | 33±1.9 | 0.4±0.2 | <0.0001 |
| $35,000 to $49,999 | 17±0.7 | 21±1.2 | 31±1.8 | 6.7±0.7 | <0.0001 |
| $50,000 to $74,999 | 20±0.7 | 9.7±0.9 | 11±1.2 | 33±1.2 | <0.0001 |
| $75,000 or more | 28±0.8 | 3.5±0.6 | 0 | 60±1.3 | <0.0001 |
| **Other** |  | | | |  |
| Anyone in household on SNAP? | 26±0.8 | 54±1.4 | 28±1.8 | 2.1±0.4 |  |

^1^ Values are percent of respondents ± SE for all variables except age, which is number of respondents. SNAP = Supplemental Nutrition Assistance Program

^2^ *P*-values reflect the test of difference among all three subgroups (2 degrees of freedom test). Adjusting for multiple tests (15), the results are still significant at *P*<0.02.

^3^ Total N for first 24-h dietary recall was 3,235, of which 6 were missing child WIC status and are not included in the analyses in this paper.

^4^ Most (92%) of WIC participants reported incomes consistent with the WIC income eligibility threshold for their reported household size, but a few did report higher incomes for which even the lower end of the reported income range is over the applicable WIC eligibility threshold. This inconsistency is a limitation of surveys relying on self-reporting. The imputation that assigns nonparticipants to “lower income” or “higher income” enforces the correct relationship among income bracket, household size, and WIC income eligibility thresholds; the lower-income nonparticipants reporting incomes over $50,000 have household sizes between 5 (eligibility threshold is $52,614) and 8 (eligibility threshold is $75,647).

Supplemental Table 2. Consumption of complementary foods, infants 6-8.9 and 9-11.9 mo, by WIC participation^1^

|  | **Child’s Age, mo** | | | | | | | | |
| --- | --- | --- | --- | --- | --- | --- | --- | --- | --- |
| **Food Category** | **6-8.9** | | | |  | **9-11.9** | | | |
|  | **All (n=468)** | **WIC participants (n=211)** | **Nonparticipants** | |  | **All (n=433)** | **WIC participants (n=164)** | **Nonparticipants** | |
|  |  |  | **Lower Income (n=69)** | **Higher Income (n=188)** |  |  |  | **Lower Income (n=100)** | **Higher Income (n=169)** |
| Milk (not breast milk or formula)^3^ | 4.1 | 4.9 | 3.4 | 2.8 |  | 17 | 15 | 26 | 15 |
| Milk products^4^ | 6.5 | 4.4 | 8.1 | 10 |  | 23 | 23 | 17 | 26 |
| Grains | 77 | 77 | 72 | 78 |  | 91 | 92 | 86 | 91 |
| Infant cereals^5^ | 55 | 56 | 47 | 57 |  | 48 | 56 | 32 | 41 |
| Fruit & fruit juices | 74 | 76 | 75 | 69 |  | 84 | 81 | 89 | 89 |
| Solid fruit^6^ | 70 | 72 | 73 | 65 |  | 78 | 74 | 83 | 86 |
| 100% juice^7^ | 22 | 29 | 12 | 12 |  | 33 | 39 | 29 | 19 |
| Vegetables^8^ | 76 | 78 | 69 | 73 |  | 68 | 69 | 56 | 72 |
| Meats/proteins^9^ | 23 | 25 | 31 | 18 |  | 52 | 50 | 48 | 60 |

^1^ Values are mean percentage of children consuming the food category during a single 24-h recall.

^2^ Lower income nonparticipants are likely WIC eligible; higher income nonparticipants are likely not WIC eligible. See the Methods section for further details.

^3^ Includes cow’s milk, plant-based substitutes, and goat’s milk, but excludes breast milk and infant formula.

^4^ Includes cheese and yogurt.

^5^ Includes any kind of infant cereal, regardless of grain (i.e., rice, oat, quinoa, wheat, multigrain, or unknown grain).

^6^ Includes both baby-food fruit and non-baby-food fruit; excludes 100% juice.

^7^ Includes both baby 100% juice and non-baby 100% juice.

^8^ Includes baby-food vegetables, non-baby-food vegetables, and white potatoes.

^9^ Includes meats, poultry, fish, legumes, nuts, seeds, but does not include cheese or yogurt.
